# Dermoscopy of Chronic Radiation-Induced Dermatitis in Patients with Head and Neck Cancers Treated with Radiotherapy

**DOI:** 10.3390/life14030399

**Published:** 2024-03-18

**Authors:** Aleksandra Pilśniak, Anastazja Szlauer-Stefańska, Andrzej Tukiendorf, Tomasz Rutkowski, Krzysztof Składowski, Grażyna Kamińska-Winciorek

**Affiliations:** 1 Department of Internal Medicine, Autoimmune and Metabolic Diseases, Faculty of Medical Sciences, Medical University of Silesia, 40-055 Katowice, Poland; aleksandra.pilsniak@gmail.com; 2 Department of Bone Marrow Transplantation and Onco-Hematology, Maria Sklodowska-Curie National Research Institute of Oncology (MSCNRIO), 44-102 Gliwice, Poland; anastazja.szlauer@gmail.com; 3 Institute of Health Sciences, Opole University, 45-040 Opole, Poland; andrzej.tukiendorf@gmail.com; 4 Inpatient Department of Radiation and Clinical Oncology, Maria Sklodowska-Curie National Research Institute of Oncology (MSCNRIO), Gliwice Branch, 44-102 Gliwice, Poland; tomasz.rutkowski@gliwice.nio.gov.pl (T.R.); krzysztof.skladowski@io.gliwice.pl (K.S.); 5 Department of Bone Marrow Transplantation and Onco-Hematology, Skin Cancer and Melanoma Team, Maria Sklodowska-Curie National Research Institute of Oncology (MSCNRIO), 44-102 Gliwice, Poland

**Keywords:** chronic radiation-induced dermatitis, radiotherapy, head and neck cancers, dermoscopy, side effects

## Abstract

Radiotherapy (RT) is an integral part of many cancer treatment protocols. Chronic radiation-induced dermatitis (CRD) is a cutaneous toxicity that occurs in one-third of all patients treated with this method. CRD is usually observed several months after completion of treatment. Typical symptoms of CRD are telangiectasia, skin discoloration, atrophy, thickening, and cutaneous fibrosis. There are currently no data in the literature on the evaluation of the dermoscopic features of CRD. The aim of this prospective study was the identification of clinical and dermoscopic features in a group of 32 patients with head and neck cancer (HNC) in whom CRD developed after RT. CRD was assessed at 3, 6, and 12 months after RT in 16, 10, and 10 patients, respectively. CRD was assessed at one time point and two time points in 28 and 4 patients, respectively. The control included skin areas of the same patient not exposed to RT. The dataset consisted of 36 clinical and 216 dermoscopic photos. Clinical evaluation was performed according to the RTOG/EORTC radiation-induced dermatitis scale. The highest score was grade 2 observed in 21 patients. Clinical observations revealed the presence of slight and patchy atrophy, pigmentation change, moderate telangiectasias, and some and total hair loss. Dotted vessels, clustered vessel distribution, white patchy scale, perifollicular white color, white structureless areas, brown dots and globules, and white lines were the most frequently noted features in dermoscopy. Three independent risk factors for chronic toxicity, such as age, gender, and surgery before RT, were identified. The dermoscopic features that had been shown in our study reflect the biological reaction of the skin towards radiation and may be used for the parametrization of CRD regarding its intensity and any other clinical consequences in the future.

## 1. Introduction

According to estimates by the American Cancer Society, more than 2 million new cases of cancer will be diagnosed in the USA in 2024. This estimated number of new cases of cancers excludes squamous and basal cell carcinoma of the skin, and cancer in situ (non-invasive cancer) [1].

Surgery, radiotherapy (RT), chemotherapy (CHT), and combinations of these methods in patients with head and neck cancer (HNC) depend on both patient- and tumor-related factors [2,3].

While RT is a crucial part of many cancer treatment protocols [4], preventing side effects, long-term disability, and discomfort from this therapy is important. The side effects mentioned above can affect the patient’s quality of life to such an extent that treatment is interrupted, which impairs the effect of RT. This problem particularly concerns patients in whom the skin is involved in irradiated areas as in HNC [5,6]. Chronic radiation-induced dermatitis (CRD) is an important and increasingly common problem because it occurs in one-third of patients after RT [7]. CRD is a cutaneous toxicity that appears several months after RT [8,9].

Typical cutaneous symptoms of CRD include telangiectasia, discoloration, skin atrophy, thickening, and cutaneous fibrosis [10,11,12]. In the course of CRD, there is histologic evidence of reduced microvascular network density and changes in blood vessel morphology [13]. Ionizing radiation activates cytokine cascades and fibrous inflammatory pathways at the molecular level, which can progress over many years and lead to significant fibrosis [14].

Dermoscopic examination combines clinical and pathological examination [15]. There are currently no data in the literature on the evaluation of the dermoscopic features of CRD. The use of dermoscopy in the assessment of CRD is an innovative approach. In the future, it may facilitate clinical assessment and thus make appropriate decisions about the further management of a patient with developed chronic skin toxicity. The evaluation of clinical and dermoscopic features in the course of CRD was the main assumption of this study.

## 2. Materials and Methods

The clinical and dermoscopic evaluation in the CRD study is a continuation of our earlier published cutaneous toxicity study in patients with HNC who are eligible for RT [16]. The first study focused on acute radiation dermatitis (ARD). However, this study looks at CRD, and although the study group is different, it is a continuum of the assessment of skin toxicity following RT. The location of the study, the methodology, the treatment protocols for the included patients, the inclusion and exclusion criteria, the clinical and dermoscopic assessment, and the statistical methods used are consistent with the first study on ARD [16].

### 2.1. Patients

The study was conducted at the Maria Skłodowska-Curie National Institute of Oncology, Gliwice Branch (MSCNRIO), from September 2020 to March 2021. The study group consisted of 32 patients who underwent RT treatment at the MSCNRIO and were under the care of the hospital oncology clinic. The observational group consisted of patients with oral cavity cancer, oropharyngeal cancer, hypopharyngeal cancer, laryngeal cancer, and nasopharyngeal cancer in 11, 7, 3, 7, and 2 cases, respectively. There was also 1 patient with sphenoid sinus cancer and 1 patient with a primary unknown tumor involved in this group. The study included 32 patients (inclusion criteria: signed informed consent, age > 18 years, radical RT) with a mean age of 60 years (range: 30–75 years). There were 10 women and 22 men in this group. Patients with active dermatoses or patients being treated with biological drugs were excluded from the study.

### 2.2. Treatment

The treatment protocol for the patients included in this study was consistent with a previously published ARD study [16]. Induction chemotherapy (indCHT) was carried out in 7 cases. Radiochemotherapy (CHRT) was performed simultaneously in 18 patients, and only RT was performed in 14 patients. Clinical target volume 1 (CTV1) included a primary tumor and involved lymph node groups. Clinical target volume 2 (CTV2) included areas at risk of harboring microscopic disease and elective lymph node groups. All patients were treated with a dose of 70 Gy, divided into 35 fractions (2.0 Gy/fraction), over 7 weeks or 70.2 Gy, divided into 39 fractions (1.8 Gy/fraction), over 5.5 weeks towards the primary target. The dose for the elective target was 50 Gy in 25 fractions (2.0 Gy/fraction) or 54 Gy in 30 fractions (1.8 Gy/ fraction). IndCHT was given as 2 to 3 cycles of TPF (docetaxel 75 mg/m^2^, cisplatin 75 mg/m^2^, d1, and 5-fluorouracil 750 mg/m^2^ d1–5) or PF (cisplatin 100 mg/m^2^, d1 and 5-fluorouracil 1000 mg/m^2^ d1–5). CHRT included RT with 2–3 cycles of concomitant cisplatin given at a dose of 100 mg/m^2^ each 21 days.

### 2.3. Clinical and Dermoscopic Evaluation

During each observation of the study, 4 dermoscopic images of the irradiated area (right cervical area, left cervical area, right submandibular area, left submandibular area) and 2 images of the control non-irradiated area (right and left lower part of retroauricular area) were obtained. The control areas (not exposed to RT) were within the same patients. The areas selected as controls were the most similar in thickness and texture to the skin on the neck and mandible. The lower part of the retroauricular area (specifically, the area behind the earlobe) is the area of the skin closest to the neck but not exposed to RT, with very similar UV exposure.

The collection consisted of 36 clinical and 216 dermoscopic photos. Of this set, 144 photos showed areas treated with RT. CRD was assessed at one time point and two time points in 28 and 4 patients, respectively. In total, 36 observations in 32 patients were collected.

Criteria for regular follow-ups in a structured form were checked at 3, 6, and 12 months after the end of RT. In total, 16 patients (96 dermoscopic images) were evaluated 3 months after RT, 10 patients (60 dermoscopic images) were evaluated 6 months after RT, and 10 patients (60 dermoscopic images) 12 months after RT. Radiation-induced dermatitis was clinically evaluated from grades 1 to 5 according to the RTOG/EORTC scale [17].

The first author performed a dermoscopic assessment each time using a DermLiteFoto dermatoscope (3Gen, LLC, San Juan Capistrano, CA, USA) at tenfold magnification. In the next step, the archived dermoscopic images were described by 2 independent dermoscopists (A.P. and A.S.-S.) blinded to patient/protocol data. Analysis of 31 dermoscopic features, according to the International Dermoscopic Society (IDS) consensus of non-neoplastic diseases, was performed [18]. Our description includes morphology and distribution of vessels, color and distribution of scale, follicular findings such as follicular plugs, follicular red dots, perifollicular white color, follicular pigmentation, as well as color and morphology of other structures, and specific clues [16,18]. In the event of disagreement between the dermoscopists, the final decision on the description was made by a third dermoscopist (G.K.-W.).

### 2.4. Statistical Analysis

In the final statistical evaluation, a database based on archived and described 216 dermoscopic images and 36 clinical images was analyzed. The degree of agreement between 2 independent researchers and the degree of agreement between the clinical and dermoscopic features was assessed using Cohen’s kappa coefficient. Univariate binary logistic regression was applied to assess the influence of RT fractions on the binary outcomes of skin diagnosis. A multivariate ordinal logistic model was used to estimate the influence of the collected risk factors on the dermoscopic images and clinical features, consistent with the methodology of the study. Due to repeated measures with consecutive RT fractions for each patient, the regressions were extended for random effects (we fitted our data using Generalized Linear Mixed Models via Penalized Quasi-Likelihood for binomial families). The statistical results were expressed by a classical odds ratio (OR) together with a 95% confidence interval (CI 95%) and a *p*-value. The computation was performed using the R statistical platform [19] with the MASS package [20].

## 3. Results

In 82% of the dermoscopic and macroscopic image evaluations, the agreement between two independent observers based on Cohen’s κ coefficient was equal to 1.0. All 32 patients observed during the follow-up after RT developed CRD. The highest score per RTOG/EORTC [17] was grade 2 observed in 21 patients. The remaining patients developed grade 1 CRD. In the 3rd month of observation, 10 patients developed grade 2 CRD, and the remaining 6 developed grade 1 CRD. In the 6th month of observation, five patients developed grade 2 CRD, and the remaining two developed grade 1. In the 12th month of observation, six patients developed grade 2 CRD, and the remaining three developed grade 1. Two patients had a change in reaction grade, i.e., from 1 to 2 for one patient and from 2 to 1 for the other patient, at 3 and 6 months of follow-up, respectively.

Table 1 shows the percentage of dermoscopic features in the respective clinical grade of CRD.

**Figure 1:**
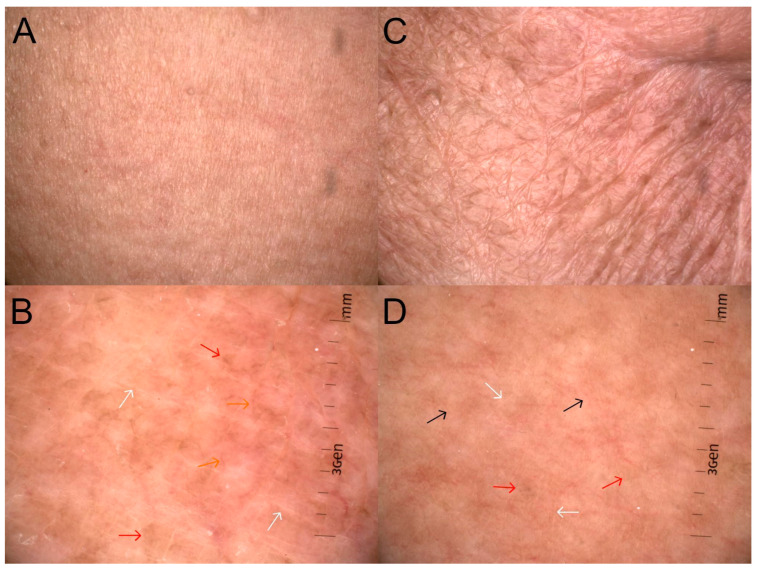
CRD on macroscopic images (**A**,**C**) across grades (G) G1 to G2, assessed according to RTOG criteria [17]. Dermoscopic images (**B**,**D**) are described according to the International Dermoscopy Society (IDS) by Errichetti et al. [18]. (**A**)—slight atrophy, pigmentation change (G1); (**B**)—linear vessels (orange arrows) in unspecific distribution, brown dots or globules (red arrows), and white lines (white arrows); (**C**)—patchy atrophy, total hair loss (G2); (**D**)—dotted vessels (black arrows) with unspecific distribution, brown dots or globules (red arrows), and white lines (white arrows).

The typical clinical and dermoscopic features are presented in Figure 1A–D.

**Figure 2:**
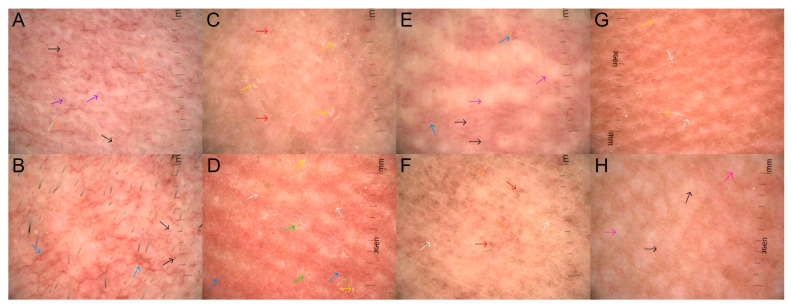
The description of the dermoscopic images of CRD based on the consensus of the experts for non-neoplastic dermatoses on behalf of the IDS [18]. (**A**)—dotted vessels (black arrows) and linear vessels (orange arrows) with unspecific distribution, and perifollicular pigmentation (purple arrows); (**B**)—dotted (black arrows) and linear with branch vessels (blue arrows) with clustered distribution; (**C**)—white patchy scale (yellow arrows), and brown dots and globules (red arrows); (**D**)—linear with branch vessels (blue arrows) with reticular distribution, perifollicular white color (green arrows), white patchy scale (yellow arrows), white lines (white arrows); (**E**)—linear with branches (blue arrows) and dotted vessels (black arrows) with clustered distribution and white structureless areas (pink arrows); (**F**)—linear vessels (orange arrows) with unspecific distribution, brown dots and globules (red arrows) and white lines (white arrows); (**G**)—white patchy scale (yellow arrows) and white lines (white arrows); (**H**)—dotted vessels (black arrows) in clustered distribution and white structureless areas (pink arrows).

The vascular polymorphisms observed in healthy skin were also found in all degrees of CRD. Of note is the significantly increased proportion of dotted vessels (Figure 2A,B,E,H), which—in healthy skin—was revealed in 18.75% of observations, while in areas subjected to RT, the incidence increased to 82.6–84.6%. The arrangement of vessels was also heterogeneous. In healthy skin, clustered distribution (Figure 2B,E,H) was evident in 18.75% of observations, in 53.9% of observations in grade 1 CRD, and 43.5% of grade 2 observations. In all degrees of CRD, yellow and brown scales were present, which was not observed in healthy skin. Moreover, the proportion of white scales (Figure 2C,D,G) compared to healthy skin (15.63%) increased to 53.9% in grade 1 CRD and to 52.2% in grade 2. Regarding follicular findings, an increase in the incidence of perifollicular white color (Figure 2D) was observed. In healthy skin, this feature was found only in 3.13% of observations, whereas in grades 1 and 2, it was noted in 15.4%, and 42.5%, respectively. With the increase in the grade of CRD, an increase in the incidence of white structureless areas (Figure 2E,H), brown dots and globules (Figure 1B,D and Figure 2C,F), and white lines (Figure 1B,D and Figure 2D,F,G) was observed. Such features were found in grade 1 in 92.3%, 69.2%, and 84.6% of patients, respectively, and in grade 2 in 100%, 95.7%, and 78.3% of patients, respectively. In healthy skin, white structureless areas (Figure 2E,H), brown dots and globules (Figure 1B,D and Figure 2C,F), and white lines (Figure 1B,D and Figure 2D,F,G) appeared in 3.13%, 31.25%, and 21.88% of patients, respectively.

Table 2 shows the relationship between dermoscopic and clinical features using the K coefficient. Statistically significant results are highlighted in bold (Table 2).

Described compatibility was 0.226–0.423 for slight atrophy, pigmentation change, thin telangiectasias, moderate telangiectasias, and dermoscopic features such as linear vessels with branches, linear curved vessels, reticular vessels, and perifollicular pigmentation.

In the next phase of the analysis, the influence of age, gender, indCHT, concomitant CHT, total radiation dose, fractional dose, tumor location, and histopathological diagnosis on the tumor size and the presence of dermoscopic and clinical features were investigated. This analysis was performed using logistic regression.

Table 3 shows statistically significant dermoscopic and clinical features and possible risk factors for CRD using odds ratios, whereas OR is a measure of association between radiation exposure and the clinical outcome; OR > 1 indicates the increased occurrence of any event, while OR < 1 indicates protective exposure.

We observed a relationship between the presence of white structureless areas (Figure 2E,H) and age, fraction dose, number of fractions, and total dose. The statistical interpretation of the OR (univariate regression) may be as follows: a 5-year difference in the age of patients generates a (1.20^5) × 100% = 249% higher risk in the occurrence of white structureless areas (Figure 2E,H). An increase of 5 fractions generates a (1.15^5) × 100% = 201% higher risk in the occurrence of white structureless areas (Figure 2E,H). A 10 Gy difference in the total dose generates a (1–1.20^10) × 100% = 137% higher risk in the occurrence of white structureless areas (Figure 2E,H). The other results in the table are to be interpreted in a similar way.

Age was a significant factor for patchy scale (Figure 2C,D,G), perifollicular pigmentation (Figure 2A), and white structureless areas (Figure 2E,H) in a univariate analysis.

Gender is an important individual factor for the presence of linear vessels (Figure 1B and Figure 2A,F) and pigmentation change (Figure 1A). The risk of linear vessels (Figure 1B and Figure 2A,F) is 94% lower for women than men, while pigmentation change (Figure 1A) is almost 1077% (almost 11 times) higher for women than men.

The presence of surgery increases the risk of the occurrence of white scale (Figure 2C,D,G) more than 4 times.

As the number of fractions and the total dose increases, the risk of white structureless areas (Figure 2E,H), brown dots and globules (Figure 1B,D and Figure 2C,F), pigmentation change (Figure 1A), as well as some and total hair loss (Figure 1C) increases. Not surprisingly, the number of fractions and total dose are also related to development grades per RTOG/EORTC [17].

Concurrent CHT increases the risk of complete hair loss by more than 6 times (OR = 6.20 (1.59–28.18)).

In the next step, as shown in Table 4, the influence of time on the occurrence of clinical and dermatoscopic characteristics was included in the analysis due to the different observation periods (Table 4).

Time in a month statistically generates a higher probability of the appearance of dotted vessels (Figure 2A,B,E,H), white scale (Figure 2C,D,G), patchy scale (Figure 2C,D,G), perifollicular white color (Figure 2D), white structureless areas (Figure 2E,H), brown dots and globules (Figure 1B,D and Figure 2C,F), and white lines (Figure 1B,D and Figure 2D,F,G).

## 4. Discussion

CRD is a subset of side effects of RT that can develop after treatment [8]. According to the literature, CRD occurs in one-third of all patients up to at least 10 years after RT [7]. Other authors believe that we cannot be precise on the time of its occurrence [21]. In our study, all observed patients developed CRD after RT. CRD is often a permanent, progressive, and potentially irreversible complication. In contrast to ARD, CRD does not heal on its own and can persist indefinitely [22].

Looking at the results of our study, in each month of observation (3rd, 6th, and 12th), grade 2 prevailed and it was the highest achieved grade among the observed patients.

In the current literature, dermoscopy has not been used to describe CRD and only clinical features have been described [23]. In the published data, we could not find a description involving the severity of CRD with dermoscopic observation. Dermoscopy assessment allows for the correlation of the macroscopic and dermoscopic features and therefore expands our knowledge about the presence and evolution of CRD. It is a much more accurate examination method than the unaided eye to confirm our diagnosis and can also result in a list of different diagnoses. Understanding the biology of dermoscopic changes observed during long-term follow-up may indicate prognostic factors for reaction severity and predictive factors for effective future treatments.

The basic mechanism for the development of chronic skin reactions is a persistent inflammatory response that begins after the first RT session and then continues for months or years [21,24].

Dotted vessels can be seen on inflamed or damaged skin [25], as confirmed by our study, which revealed an over fourfold increase in the incidence of this type of vessels in skin with diagnosed CRD compared to healthy skin. Moreover, clustered vessels can also be seen in dermatitis, and this is due to vasodilation in focally elongated dermal papillae [18,25]. The incidence of this type of distribution increased twofold in the skin with developed CRD compared to healthy skin in our results. The white scale, which is a feature of dermatoses characterized by hyperkeratosis (especially parakeratosis) without serous exudate, was also observed twice as often in CRD [18]. A greater incidence of perifollicular white color with a higher degree of CRD was noted. Histologically, perifollicular white color may correspond to perifollicular fibrosis [18].

According to our first study on ARD [16], we also attempted to investigate the correlation between clinical and dermoscopic features of CRD. The macroscopic features corresponded with dermoscopic features such as linear vessels with branches, linear curved vessels, vessels with reticular distribution, vessels with unspecific distribution, perifollicular white color, white structureless areas, and perifollicular pigmentation.

Radiation dermatitis can occur as a result of occupational or accidental exposure to ionizing radiation [26]. The risk factors for this disorder can be classified as intrinsic, extrinsic, or both.

Intrinsic factors include age, gender, smoking, nutritional status, genetic factors, connective tissue and skin diseases, concurrent CHT, and targeted therapy [27]. Extrinsic factors, on the other hand, are mainly related to the type and dose of radiation received [28].

Meyer et al. [29] showed that the risk of late severe toxicity increases with age. In our data, age was a significant factor for patchy scale and white structureless areas, the frequency of which increases in CRD compared to healthy skin. Moreover, gender also had a significant impact on the development of CRD [29]. In our study, women were more than 11 times more likely to have a pigmentation change. An increase in the number of fractions and the total dose raises the risk of white structureless areas, brown dots and globules, pigmentation change as well as some and total hair loss.

Not surprisingly, the number of fractions and total irradiation dose are also related to the development of higher grades per RTOG/EORTC. According to the previous study by Kawamura et al. [30], concomitant CHT is important for the development of ARD.

Toledano et al. in their study found that after breast-conserving surgery in patients, the simultaneous use of adjuvant CHT and RT increases late toxicity [31]. In our study, concurrent CHT increased the risk of complete hair loss by more than 6 times.

### Limitations of the Study

The study group in our prospective observational study included a cohort of patients undergoing RT due to HNC. Despite the significant prevalence of this malignancy and the prevalent ARD in the course of this RT procedure, it should be emphasized that the small group of patients is due to the rarer occurrence of CRD and the longer follow-up time is required for its diagnosis and the unfavorable prognosis of treated patients, resulting in the possibility of losing patients from follow-up.

Criteria for regular follow-ups in a structured form were checked at 3, 6, and 12 months after the end of RT. However, the lack of regular follow-ups was mainly due to the loss of patients in this group with an unfavorable prognosis. In our study, only four patients were followed at two time points.

Another limitation of this study is the absence of cohorts of patients who do not develop CRD. Such a control group should be included in further research, or the group size should be increased by including subsequent patients after RT, regardless of clinically expressed reaction.

## 5. Conclusions

This is the first study showing the potential role of dermoscopy in CRD evaluation. Until now, there was no other objective tool for qualitative analyses of CRD. The dermoscopic features that had been shown in our study reflect the biological reaction of the skin towards radiation and may be used for the parametrization of CRD regarding its intensity and any other clinical consequences in the future. However, further research is needed to confirm the role of dermoscopy and to gather more clinical data on its utilization in CRD assessment.

## Figures and Tables

**Table 1:** The proportion of dermoscopic features, based on the consensus of the experts for non-neoplastic dermatoses on behalf of the IDS [18], to the grade of CRD according to the RTOG/EORTC scale [17]. The proportion was based on the analysis of 216 dermoscopy images in 6 areas (4 irradiated areas and 2 control areas) in 32 patients (in 28 patients with one time point and 4 patients with two time point observations).

		CRD	
RTOG/EORTC	0	Grade 1	Grade 2
Vessel morphology			
Dotted	18.75%	84.6%	82.6%
Linear (without bends or branches)	3.13%	7.7%	4.4%
Linear with branches	65.63%	61.5%	69.6%
Linear curved	46.88%	76.9%	47.8%
Vessel distribution			
Uniform	0.0%	0.0%	0.0%
Clustered	18.75%	53.9%	43.5%
Peripheral	0.0%	0.0%	0.0%
Reticular	0.0%	0.0%	4.4%
Unspecific	75.0%	84.6%	91.3%
Scale color			
White	15.63%	53.9%	52.2%
Yellow	0.0%	7.7%	13.0%
Brown	0.0%	15.4%	17.4%
Scale distribution			
Diffuse	0.0%	0.0%	0.0%
Central	0.0%	0.0%	0.0%
Peripheral	0.0%	7.7%	0.0%
Patchy	15.63%	46.2%	56.5%
Follicular findings			
Follicular plugs	0.0%	0.0%	0.0%
Follicular red dots	0.0%	0.0%	0.0%
Perifollicular white color	3.13%	15.4%	43.5%
Perifollicular pigmentation	9.38%	23.1%	0.0%
Other structures			
White structureless	3.13%	92.3%	100.0%
Brown structureless	0.0%	0.0%	0.0%
Yellow structureless	0.0%	0.0%	0.0%
White dots or globules	0.0%	0.0%	0.0%
Brown dots or globules	31.25%	69.2%	95.7%
Yellow dots or globules	0.0%	0.0%	0.0%
White lines	21.88%	84.6%	78.3%
Brown lines	0.0%	0.0%	0.0%
Yellow lines	0.0%	0.0%	0.0%

**Table 2:** The degree of agreement between the presence of selected clinical [17] and dermoscopic features [18] in CRD was assessed with values of κ statistics.

Dermoscopic Features	Clinical Features						
	Slight Atrophy	Patchy Atrophy	Pigmentation Change	Some Hair Loss	Total Hair Loss	Thin Telangiectasias	Moderate Telangiectasias
Dotted vessels	−0.05	0.0625	0.0357	0.111	−0.111	0.16	−0.0125
Linear vessels	0.0217	−0.0161	0.0188	0.111	−0.111	−0.0112	−0.0862
Linear vessels with branches	−0.105	0.118	0.0488	0	0	**0.423**	0.149
Linear curved vessels	0.135	−0.143	−0.0105	0.278	−0.278	0.264	**0.207**
Clustered vessels	0.103	−0.101	−0.0682	0.167	−0.167	0.0613	0.19
Reticular vessels	−0.0549	0.04	0.00917	0.0556	−0.0556	−0.0559	**0.301**
Unspecific vessels	−0.0328	0.0426	**0.366**	0	0	0.027	0.0395
White scale	−0.101	0.103	0.074	0.0556	−0.0556	0.161	0.145
Yellow scale	−0.0851	0.0656	0.0395	0	0	0.0795	−0.141
Brown scale	−0.0625	0.05	−0.0125	0.111	−0.111	0.069	0.0357
Peripheral scale	0.0769	−0.056	0.00917	0.0556	−0.0556	0.0447	−0.0485
Patchy scale	−0.211	0.215	0.074	0.0556	−0.0556	0.0497	0.145
Perifollicular white color	**−0.353**	**0.316**	0.149	0	0	−0.0714	−0.0976
Perifollicular pigmentation	**0.226**	**−0.171**	**−0.11**	0.167	−0.167	0.136	−0.116
White structureless areas	−0.06	0.08	**0.30**	−0.06	0.06	0.07	0.01
Brown dots or globules	−0.19	0.24	0.30	−0.17	0.17	−0.15	−0.02
White lines	0.09	−0.11	0.01	0.06	−0.06	−0.25	0.00

**Table 3:** The influence of clinical data on the occurrence of macroscopic and dermoscopic features in CRD. The table shows statistically significant OR (*p* < 0.05) (in univariate binary logistic regression).

Dermoscopic Features		Univariate Analysis (OR (95%CI) *p*-Value)
Linear vessels (without bends or branches)	Gender	0.06 (0.00–0.89) 0.0400
White scale	Operation	4.06 (1.06–17.68) 0.0402
Patchy scale	Age	1.10 (1.01–1.23) 0.0264
Perifollicular pigmentation	Age	1.31 (1.03–1.98) 0.0243
White structureless areas	Age	1.20 (1.04–1.71) 0.0105
	Fraction dose	0.17 (0.00–0.65) 0.0092
	Number of fractions	1.15 (1.02–3.48) 0.0235
	Total dose	1.09 (1.02–1.77) 0.0168
Brown dots or globules	Number of fractions	1.10 (1.02–1.20) 0.0171
	Total dose	1.05 (1.00–1.10) 0.0339
**Clinical Features**		
Pigmentation change	Gender	11.77 (1.79–133.87) 0.0098
	Number of fractions	1.11 (1.03–1.21) 0.0088
	Total dose	1.06 (1.02–1.12) 0.0086
Some hair loss	Number of fractions	0.87 (0.59–0.97) 0.0070
	Total dose	0.84 (0.67–0.98) 0.0038
	Radiochemotherapy	0.18 (0.04–0.69) 0.0123
Total hair loss	Number of fractions	1.12 (1.02–1.45) 0.0141
	Total dose	1.16 (1.02–1.45) 0.0067
	Radiochemotherapy	6.20 (1.59–28.18) 0.0080
RTOG	Number of fractions	1.07 (1.00–1.18) 0.0479
	Total dose	1.04 (1.00–1.11) 0.0494

**Table 4:** The influence of time on the occurrence of macroscopic and dermoscopic features in CRD. The table presents only dermoscopic structures that appeared statistically significant over time, extracted from all assessed dermoscopic structures presented in CRD. The table shows statistically significant ORs (*p* < 0.05) (in univariate binary logistic regression).

Dermoscopic Features	Univariate Analysis (OR (95%CI) *p*-Value)
Dotted vessels	1.31 (1.12–1.59) 0.0002
White scale	1.18 (1.04–1.34) 0.0068
Patchy scale	1.22 (1.08–1.40) 0.0011
Perifollicular white color	1.21 (1.06–1.39) 0.0047
White structureless areas	2.67 (1.78–4.46) <0.0001
Brown dots or globules	1.82 (1.35–2.69) <0.0001
White lines	1.25 (1.09–1.50) 0.0008

## Data Availability

The data presented in this study are available upon request from the corresponding author.

## References

[1] American Cancer Society Cancer Facts & Figures 2024. https://www.cancer.org.

[2] Gamerith G., Fuereder T. (2020). Treating head and neck cancer—A multidisciplinary effort. Memo.

[3] Ziółkowska E., Biedka M., Windorbska W. (2011). Odczyn popromienny u chorych na raka regionu głowy i szyi: Mechanizmy i konsekwencje. Otorynolaryngologia.

[4] Borrelli M.R., Shen A.H., Lee G.K., Momeni A., Longaker M.T., Wan D.C. (2019). Radiation-Induced Skin Fibrosis: Pathogenesis, Current Treatment Options, and Emerging Therapeutics. Ann. Plast Surg..

[5] DeSantis C.E., Lin C.C., Mariotto A.B., Siegel R.L., Stein K.D., Kramer J.L., Alteri R., Robbins A.S., Jemal A. (2014). Cancer treatment and survivorship statistics. CA Cancer J. Clin..

[6] Dudek A., Rutkowski T., Kamińska-Winciorek G., Krzysztof Składowski K. (2020). What is new when it comes to acute and chronic radiation-induced dermatitis in head and neck cancer patients?. Nowotw. J. Oncol..

[7] Whelan T.J., Pignol J.P., Levine M.N., Julian J.A., MacKenzie R., Parpia S., Shelley W., Grimard L., Bowen J., Lukka H. (2010). Long-term results of hypofractionated radiation therapy for breast cancer. N. Engl. J. Med..

[8] Hegedus F., Mathew L.M., Schwartz R.A. (2017). Radiation dermatitis: An overview. Int. J. Dermatol..

[9] Robijns J., Laubach H.J. (2018). Acute and chronic radiodermatitis. J. Egypt. Women’s Dermatol. Soc..

[10] Wong R.K., Bensadoun R.J., Boers-Doets C.B., Bryce J., Chan A., Epstein J.B., Eaby-Sandy B., Lacouture M.E. (2013). Clinical practice guidelines for the prevention and treatment of acute and late radiation reactions from the MASCC Skin Toxicity Study Group. Support. Care Cancer.

[11] Lanigan S.W., Joannides T. (2003). Pulsed dye laser treatment of telangiectasia after radiotherapy for carcinoma of the breast. Br. J. Dermatol..

[12] Yarnold J., Brotons M.C. (2010). Pathogenetic mechanisms in radiation fibrosis. Radiother. Oncol..

[13] Phulpin B., Gangloff P., Tran N., Bravetti P., Merlin J.L., Dolivet G. (2009). Rehabilitation of irradiated head and neck tissues by autologous fat transplantation. Plast. Reconstr. Surg..

[14] Martin M., Lefaix J.L., Delanian S. (2000). TGF-β1 and radiation fibrosis: A master switch and a specific therapeutic target?. Int. J. Radiat. Oncol. Biol. Phys..

[15] Kamińska-Winciorek G., Pilśniak A. (2021). The role of dermoscopy in dermato-oncological diagnostics—New trends and perspectives. Nowotw. J. Oncol..

[16] Pilśniak A., Szlauer-Stefańska A., Tukiendorf A., Rutkowski T., Składowski K., Kamińska-Winciorek G. (2023). Dermoscopy of acute radiation-induced dermatitis in patients with head and neck cancers treated with radiotherapy. Sci. Rep..

[17] Cox J.D., Stetz J., Pajak T.F. (1995). Toxicity criteria of the Radiation Therapy Oncology Group (RTOG) and the European Organization for Research and Treatment of Cancer (EORTC). Int. J. Radiat. Oncol. Biol. Phys..

[18] Errichetti E., Zalaudek I., Kittler H., Apalla Z., Argenziano G., Bakos R., Blum A., Braun R.P., Ioannides D., Lacarrubba F. (2020). Standardization of dermoscopic terminology and basic dermoscopic parameters to evaluate in general dermatology (non-neoplastic dermatoses): An expert consensus on behalf of the International Dermoscopy Society. Br. J. Dermatol..

[19] R Core Team (2022). R: A Language and Environment for Statistical Computing.

[20] Venables W.N., Ripley B.D. (2022). Modern Applied Statistics with S.

[21] Spałek M. (2016). Chronic radiation-induced dermatitis: Challenges and solutions. Clin. Cosmet. Investig. Dermatol..

[22] Hymes S.R., Strom E.A., Fife C. (2006). Radiation dermatitis: Clinical presentation, pathophysiology, and treatment. J. Am. Acad. Dermatol..

[23] Bray F.N., Simmons B.J., Wolfson A.H., Nouri K. (2016). Acute and Chronic Cutaneous Reactions to Ionizing Radiation Therapy. Dermatol. Ther..

[24] Vozenin-Brotons M.C., Milliat F., Sabourin J.C., de Gouville A.C., François A., Lasser P., Morice P., Haie-Meder C., Lusinchi A., Antoun S. (2003). Fibrogenic signals in patients with radiation enteritis are associated with increased connective tissue growth factor expression. Int. J. Radiat. Oncol. Biol. Phys..

[25] Anscher M.S. (2005). The irreversibility of radiation-induced fibrosis: Fact or folklore?. J. Clin. Oncol..

[26] Wolff K., Johnson R., Saavedra A. (2013). Skin reactions to ionizing radiation. Fitzpatrick’s Color Atlas and Synopsis of Clinical Dermatology.

[27] Porock D. (2002). Factors influencing the severity of radiation skin and oral mucosal reactions: Development of a conceptual framework. Eur. J. Cancer Care.

[28] Hegedus F., Schwartz R.A. (2019). Cutaneous radiation damage: Updating a clinically challenging concern. G. Ital. Dermatol. Venereol..

[29] Meyer F., Fortin A., Wang C.S., Liu G., Bairati I. (2012). Predictors of severe acute and late toxicities in patients with localized head-and-neck cancer treated with radiation therapy. Int. J. Radiat. Oncol. Biol. Phys..

[30] Kawamura M., Yoshimura M., Asada H., Nakamura M., Matsuo Y., Mizowaki T. (2019). A scoring system predicting acute radiation dermatitis in patients with head and neck cancer treated with intensity-modulated radiotherapy. Radiat. Oncol..

[31] Toledano A., Garaud P., Serin D., Fourquet A., Bosset J.F., Breteau N., Body G., Azria D., Le Floch O., Calais G. (2006). Concurrent administration of adjuvant chemotherapy and radiotherapy after breast-conserving surgery enhances late toxicities: Long-term results of the ARCO-SEIN multicenter randomized study. Int. J. Radiat. Oncol. Biol. Phys..

